# Factors Influencing Continued Wearable Device Use in Older Adult Populations: Quantitative Study

**DOI:** 10.2196/36807

**Published:** 2023-01-19

**Authors:** Karla Muñoz Esquivel, James Gillespie, Daniel Kelly, Joan Condell, Richard Davies, Catherine McHugh, William Duffy, Elina Nevala, Antti Alamäki, Juha Jalovaara, Salvatore Tedesco, John Barton, Suzanne Timmons, Anna Nordström

**Affiliations:** 1 Department of Computer Science Atlantic Technological University Letterkenny Ireland; 2 Faculty of Computing, Engineering, and the Built Environment Ulster University Derry United Kingdom; 3 Department of Physiotherapy Karelia University of Applied Sciences Karelia Finland; 4 Wireless Sensors Network Group Tyndall National Institute University College Cork Cork Ireland; 5 Centre for Gerontology and Rehabilitation School of Medicine University College Cork Cork Ireland; 6 Department of Public Health and Clinical Medicine Umeå University Umeå Sweden

**Keywords:** usability, older adults, remote sensing, sensor systems, wearable device, mobile phone

## Abstract

**Background:**

The increased use of wearable sensor technology has highlighted the potential for remote telehealth services such as rehabilitation. Telehealth services incorporating wearable sensors are most likely to appeal to the older adult population in remote and rural areas, who may struggle with long commutes to clinics. However, the usability of such systems often discourages patients from adopting these services.

**Objective:**

This study aimed to understand the usability factors that most influence whether an older adult will decide to continue using a wearable device.

**Methods:**

Older adults across 4 different regions (Northern Ireland, Ireland, Sweden, and Finland) wore an activity tracker for 7 days under a free-living environment protocol. In total, 4 surveys were administered, and biometrics were measured by the researchers before the trial began. At the end of the trial period, the researchers administered 2 further surveys to gain insights into the perceived usability of the wearable device. These were the standardized System Usability Scale (SUS) and a custom usability questionnaire designed by the research team. Statistical analyses were performed to identify the key factors that affect participants’ intention to continue using the wearable device in the future. Machine learning classifiers were used to provide an early prediction of the intention to continue using the wearable device.

**Results:**

The study was conducted with older adult volunteers (N=65; mean age 70.52, SD 5.65 years) wearing a Xiaomi Mi Band 3 activity tracker for 7 days in a free-living environment. The results from the SUS survey showed no notable difference in perceived system usability regardless of region, sex, or age, eliminating the notion that usability perception differs based on geographical location, sex, or deviation in participants’ age. There was also no statistically significant difference in SUS score between participants who had previously owned a wearable device and those who wore 1 or 2 devices during the trial. The bespoke usability questionnaire determined that the 2 most important factors that influenced an intention to continue device use in an older adult cohort were device comfort (τ=0.34) and whether the device was fit for purpose (τ=0.34). A computational model providing an early identifier of intention to continue device use was developed using these 2 features. Random forest classifiers were shown to provide the highest predictive performance (80% accuracy). After including the top 8 ranked questions from the bespoke questionnaire as features of our model, the accuracy increased to 88%.

**Conclusions:**

This study concludes that comfort and accuracy are the 2 main influencing factors in sustaining wearable device use. This study suggests that the reported factors influencing usability are transferable to other wearable sensor systems. Future work will aim to test this hypothesis using the same methodology on a cohort using other wearable technologies.

## Introduction

### Background

Advancements in health care have resulted in increases in life expectancy. As a consequence, a growing proportion of the population are older adults [1]. This aging population, accompanied by an increasing number of older adults becoming physically inactive [2], is placing an additional burden on health care systems and directing research toward early detection or prevention of future medical issues. Remote rehabilitation and monitoring provide an opportunity to reduce demands on health care systems and the inevitable costs associated with providing care for an aging population [3]. Remote rehabilitation can allow for access to health-based resources such as nurses, health practitioners, and specialists through technology while avoiding associated costs such as travel [4]—from both a monetary and environmental perspective. Some technology solutions have been adapted for remote rehabilitation over the last decade. Synchronous videoconferencing, for example, is one of the most commonly used technologies to deliver rehabilitation therapy to clients who are in a different location from their therapist [5]. Wearable sensor systems have recently been used to provide insights into physical activity, physical function, and general health, and as a result, therapists and clinicians can provide more detailed insights into a patient’s health and progress on a remote basis. At present, most research on wearable sensor technology is developed with accuracy at the center of the study design. This often comes at the expense of usability [6] even though research studies have indicated that perceived ease of use is just as important as perceived usefulness when it comes to technology acceptance [7]. Indeed, Mancini and Horak [8] note that, to achieve successful adoption of remote rehabilitation technologies, the solution must be both practical and usable, which is of particular importance when considering wearable sensor systems.

The Smart Sensor Devices for Rehabilitation and Connected Health project focuses on monitoring the physical capacity of older adults. The project evaluated wireless sensor systems and their capabilities for remote rehabilitation with a particular focus on end-user acceptance. The ultimate goals of technology-assisted personal health management are both continued long-term use of the device and improved well-being [9]. This study specifically focused on understanding the factors influencing continued long-term use.

Understanding the factors that influence continued device use is important as this will inform future wearable device design, ensuring that adoption and the impact of that adoption has the highest possible chance of success. This will, in turn, allow for the successful rollout of telehealth services in the future, such as remote rehabilitation, and overall increase the likelihood of improved well-being.

### Related Work

Previous work has already shown that, for monitoring technology to be accepted by older adults, it must be easy to use and not impair mobility and independence [10]. Research has also shown that human factors such as portability and resilience are the main factors that influence continued device use [11].

Older adults are interested in smart wearable devices that offer functionality for daily living and are more likely to consider using one if compatible [12]; thus, device selection is important [13]. The user’s attributes and device features are the main characteristics to observe when evaluating wearable devices [14]. Environmental and individual features need to be considered when deciding on a sensor technology device; in particular, a device that is user focused would be valuable [15]. A wearable device offering user-friendly features for everyday tasks is more appealing to individuals as they trust the information provided. If an individual trusts the device, there is an increased chance of continued use [14]. A positive finding from previous research related to older adults using activity monitoring technology showed that older adults did not struggle to use new technology [16].

The literature shows that usability challenges must be addressed to increase the likelihood of continued device use. Therefore, technology must be designed and implemented such that it is practical, unobtrusive, and well-received by older adults and, ultimately, promotes health benefits.

Balance is one of the critical characteristics used to assess the functional capacity of older adults in the literature. However, despite the growing number of articles supporting the use of balance assessment technology, there are still substantial gaps in the full understanding of the technology. In particular, existing literature in the area does not consider factors that may affect the continued long-term use of wearable technology in real-world conditions. A previous study examined the real-world use of multiple wearable sensors, noting that participants found wrist-worn sensors to be the most favorable as they were adaptable and user-friendly [15]. Core areas to focus on are preferred features of the wearable device and possible issues arising from older adults operating the device [17]. Longitudinal studies have also been suggested as an approach when assessing the usability of wearable sensors to ascertain whether ratings change with extended device use and user experience [14].

There are limited quantitative studies on long-term device usability to observe the associated influencing factors [18]. User feedback is key to understanding participants’ experience in a study and is necessary for determining whether older adults will continue to use wearable technology [19]. A previous study focused on individual preferences and asked participants to share their experiences using a fitness device; the main issues were remembering to wear the device, lack of comfort when wearing it, limited sharing support to determine a baseline with others, and inaccurate data recorded during activities [20].

We conducted a study aimed to better understand the usability factors that most influence whether an older adult will decide to continue to use a wearable device. We hypothesized that initial perceptions related to human factors of a wearable sensor system can be used as a predictor of continued device use in the future. To test this hypothesis, a study was designed to analyze data related to older adults’ perceptions of wearable activity trackers after 7 days of use. Data were collected related to participant perceptions of wearable device human factors as well as measurements of participant functional status, health status, and wearable device activity tracker measurements from a cohort of 65 older adults aged ≥65 years. One of the objectives of the research was to assess what specific factors may affect participants’ intention to continue using the wearable device beyond the 7-day study period. There are three key methodologies that could be used for usability assessment: (1) inspection involving expert observation (eg, heuristic evaluation), (2) inquiry involving qualitative data collection (eg, surveys), and (3) testing involving quantitative data collection in a real environment (eg, remote usability testing) [13]. The methodology used in this study applies both inquiry and testing methods based on a bespoke usability questionnaire completed by participants after the 7-day study period during which they used a Xiaomi Mi Band 3.

## Methods

### Overview

This section will cover the protocol used during the data collection process and describe each of the questions asked and how they relate to measuring usability. Detailed information will also be provided for the participant cohort and the hardware used to capture their activity data. This section establishes the methods used to process and analyze the data and, finally, predict continued device use.

### Protocol

This was a retrospective case series–based study. A series of older adults aged ≥65 years were given a wearable device and observed over a 1-week period. The usability data of the wearable device were then characterized among the participant series.

The study was based on a free-living data collection protocol conducted over a 7-day period. A free-living data collection protocol is a common method of collecting data from participants, particularly in sensor-based studies. The *free-living* aspect indicates that data are collected from a participant’s normal everyday living environment, typically over a period of ≥24 hours. This approach aims to eliminate any social, behavioral, and environmental biases that would otherwise be present in other testing or simulated environments.

Participants for this study were recruited from 4 different countries within the Northern Periphery and Arctic regions of Europe. These were namely Northern Ireland, Ireland, Finland, and Sweden. Inclusion criteria required participants to be aged ≥65 years, have the physical capacity to walk 20 m without the assistance of another person, and be cognitively able to answer questionnaires.

Participants met the researchers performing the trial in person twice—once at the start of the trial and again 7 days later at the end of the trial. In the first meeting, the researchers took body measurements (height, weight, and grip strength for both hands), trained the participants in device use using a standardized training manual, and asked them to complete 4 health-related questionnaires. Participants were then asked to complete two physical function tests: (1) the Five-Time Sit-to-Stand test (STS5) and (2) two 10-m walk tests. For the STS5 test, the total time to complete 5 repetitions of going from a seated position to a standing position was recorded. For the 10-m walk test, the time to complete each of the two 10-m walks was recorded (WT10M1 and WT10M2) as well as the number of steps taken in each walk (WS10M1 and WS10M2).

**Figure 1:**
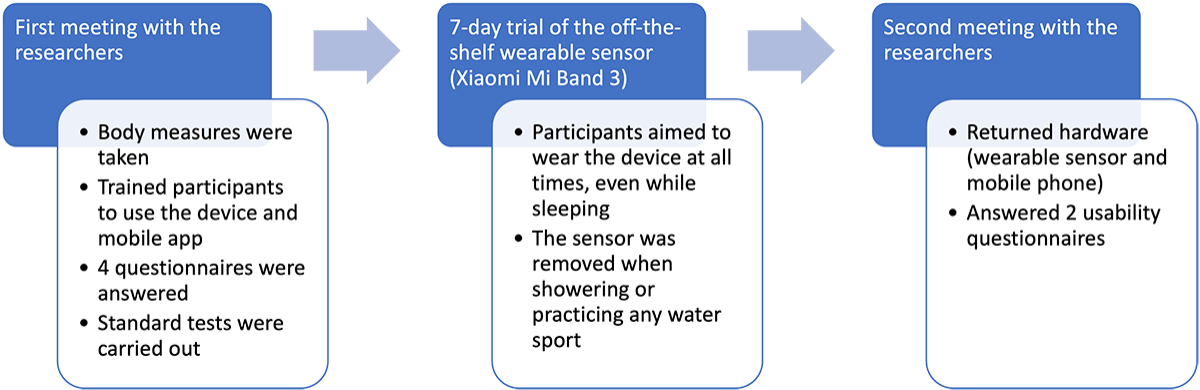
Flowchart of the study protocol.

A date for the second meeting was agreed upon, and participants were given the wearable device to wear and bring home with them. At the second meeting, the participants met with the researcher and returned the wearable device. The participants were then asked to complete 2 posttrial questionnaires focusing on usability and human factors. Figure 1 provides an overview of the study process. Ethics approval for the research study was granted at each of the 4 test sites (Ulster University, United Kingdom; Tyndall National Institute, University College Cork, Ireland; Umeå University, Sweden; and Karelia University of Applied Sciences, Finland).

The standard tests (eg, sit-to-stand and timed walk) and measures that were carried out as part of this study were used to assess the physical health of the participants. Although this study focused on usability and intention to continue using the device, data from the standard tests are part of a larger study, and further data analyses will be performed for potential future publication.

### Questionnaires

The trial used 6 questionnaires to collect various data from the participants. A total of 4 questionnaires were administered before the trial began, and 2 were administered after trial completion. The four *Pretrial Questionnaires* were as follows: (1) the 36-item Short Form Health Survey (SF-36) [21], a set of generic, coherent, and easily administered quality-of-life measures; (2) the Mini-Mental State Examination (MMSE) [22], a set of questions used to assess a patient’s cognitive impairment; (3) the Geriatric Depression Scale (GDS) [23], used to assess depressive symptomatology in older adults; and (4) the Mobile Device Proficiency Questionnaire (MDPQ) [24], used to accurately assess the mobile device proficiency of older adults. Each questionnaire was administered using an interview-based approach where the researcher asked the participants each question and indicated the possible answers they could give. The participants provided a verbal answer to each question, and the researcher recorded the results on paper and, subsequently, electronically. The questionnaires at the first meeting were administered in the following order: (1) SF-36, (2) MMSE, (3) GDS, and (4) MDPQ.

The SF-36 questionnaire was used to understand the participants’ general health status. The MMSE and GDS questionnaires were used to understand the mental health status of the participants. A score of <25 on the MMSE indicates some degree of dementia, whereas a score of >4 on the GDS indicates some degree of depression. The data were used to characterize the overall health of the participant group and understand the relative health of the participants compared with the general population. The MDPQ was used to estimate their level of familiarity and experience using technology and whether this was linked to continued device use afterward. Questionnaire scores and statistics are discussed in the *Results* section.

The two *Posttrial Questionnaires* were as follows: (1) the System Usability Scale (SUS) [25], used as a standardized method to evaluate the usability of wearable devices and facilitate benchmarking with other studies, and (2) the usability questionnaire, a bespoke questionnaire designed by the research team to specifically understand older adults’ opinions on wearable sensor technology (Multimedia Appendix 1). This usability questionnaire gathered user opinions on perceived usefulness, comfort, and ease of use as previous research studies have indicated that these are the crucial factors that influence the usability of a wearable device and can ultimately affect the likelihood of continued long-term use [26-28]. Each of these questionnaires was administered using an interview-based approach following the same methodology as the pretrial meeting. The participants were first administered the SUS questionnaire, followed by the bespoke usability questionnaire.

The SUS is a standardized and validated short 10-question survey to help validate the usability of a piece of hardware, software, or wearable device. However, to better understand the participants’ specific opinions of the wearable device usability, a bespoke usability questionnaire was designed, entitled “Accuracy, feasibility and acceptability of wireless monitoring in older people.” The questionnaire first collected dichotomous data on the participants’ familiarity with wearable devices and whether they liked the appearance. Then, a series of questions related to usability, accuracy, and acceptability were asked using an ordinal 5-category scale ranging from *strongly agree* to *strongly disagree*. The questionnaire ended with 4 general questions that gathered data on length of time worn and use at night. The final and most important question regarding this study asked the participants if they would continue to use the device after the trial had finished. Responses to this final question were analyzed to gain further insights into what factors influence the intention to continue using the device.

The bespoke usability questionnaire was designed as part of the European Union Interreg Northern Periphery and Arctic Smart Sensor Devices for Rehabilitation and Connected Health project. Experts on this project—who were from clinical, physiotherapy, and technological backgrounds in Sweden, Finland, Ireland, and Northern Ireland—worked together in a workshop meeting that was held in May 2019 in Derry/Londonderry to propose, agree, and finalize a set of questions appropriate to assess the different human factors associated with the wearable sensor system. The bespoke questionnaire was applied for the first time in this study.

### Hardware and Software

Each participant in the study was provided with a Xiaomi Mi Band 3 activity tracker, which was secured on the wrist of their nondominant hand. In addition, the participant was provided with a Huawei Y6 smartphone to facilitate interaction with the activity tracker software. Anonymous Google accounts were created to capture the activity data from each participant. Approximately half (37/65, 57%) of the participants were also requested to wear an Axivity AX3 wrist-worn accelerometer on their dominant hand. The initial plan was to have all participants wear 2 trackers as the raw data collection capabilities of the Axivity AX3 would have facilitated benchmarking between potentially new algorithms and the Xiaomi Mi Band 3. However, an initial feasibility study conducted in Finland on a small number of participants used the bespoke usability questionnaire to conclude that, generally, participants reported usability issues because of wearing 2 trackers. To help keep this from becoming an issue, it was decided to only allow half of the participants to wear 2 trackers. The distribution of those wearing 2 trackers versus 1 was approximately 50% across all the sites (Sweden: 9/20, 45%; Finland: 13/23, 57%; Northern Ireland: 7/14, 50%; Ireland: 8/8, 100%) except for the 100% (8/8) of participants at the site in Ireland who wore 2 trackers. Unfortunately, because of the unexpected impact of COVID-19 in March 2020, the trial in Ireland was interrupted midway, which resulted in 40% (8/20) of the participants receiving 2 trackers and the remaining 60% (12/20) of the participants being unable to take part.

### Cohort Description

In total, 65 participants from the 4 locations took part in the study. The mean age of the participants was 70.52 (SD 5.65) years. The mean height of the population was 169.43 (SD 9.05) cm, and the mean weight was 73.45 (SD 13.09) kg. The cohort comprised 57% (37/65) women and 43% (28/65) men. A total of 91% (59/65) of the participants were right-handed, and 9% (6/65) were left-handed. The participants were recruited using leaflets and posters. Recruitment sought older adult volunteers wanting to experience the use of wearable technology such as activity trackers in their daily lives. They should be physically able to walk 20 m unaided and cognitively able to answer questionnaires. The participants were to have no underlying health conditions other than frailty. Targeted recruitment focused on recruiting participants in community centers focusing on older adults (Eglinton Community Centre, Old Library Trust Healthy Living Centre, and U3AFoyle, all in Northern Ireland) and clinics (in Ireland, Sweden, and Finland).

### Data Processing, Analysis, and Classification

#### Overview

An analysis was performed on the collected data to understand the usability factors that most influence whether an older adult will decide to continue using a wearable device. The analysis was divided into four main areas: (1) cohort characteristic analysis, (2) SUS analysis, (3) bespoke usability questionnaire analysis, and (4) predictive modeling. The following sections describe the methods used for each of these areas.

#### Cohort Characteristic Analysis

Statistical analysis of participants’ demographics, health status, and selected usability results was performed to provide information on the characteristics of the cohort being analyzed in further sections. The cohort of 65 people comprised volunteers from Northern Ireland (n=14, 22%), the Republic of Ireland (n=8, 12%), Finland (n=23, 35%), and Sweden (n=20, 31%). For each participant, a set of 69 features were recorded. The features comprised body metrics, functional test measures, wearable device data, and questionnaire results.

Analysis of the data was performed using SPSS (version 26; IBM Corp) and the Spyder Python integrated development environment (version 5.1.5). Statistical analyses were performed using the Kendall τb, Pearson, or Spearman correlations where appropriate. The analysis also involved computing features such as the mean, variance, and SDs as well as exploring frequencies, histograms, distributions, and statistical tests.

#### SUS Analysis

Participants were asked to answer the SUS questionnaire to evaluate the Xiaomi Mi Band 3 activity tracker after an average device use of 7.12 (SD 1.53) days. Only the usability of the wearable device was to be considered by the participants.

SUS scores were analyzed to investigate whether geographical location, sex, number of wearables used, or age affected the usability rating. For our analysis, the participants who took part in the trial from Northern Ireland and the Republic of Ireland were grouped into 1 cohort comprising 22 participants because of the relatively small sample size (8/65, 12%) available for the Republic of Ireland and because of their geographical proximity, encompassing the island of Ireland. For the age analysis, we created 3 bins (<70 years, between 70 and 74 years, and >74 years) and categorized the participants accordingly.

The analysis of the SUS data aimed to understand whether geographic location, age, sex, or number of devices worn had an influence on the perceived usability of the device.

#### Bespoke Usability Questionnaire Analysis

Analysis of data from the bespoke questionnaire focused on understanding responses to question 21: “Would you continue to use the device and app again after the trial is finished?” Various analyses were performed on this question to gain insights into what factors influence continued device use. Analysis of the statistical distributions of participants from the 2 groups (participants who indicated that they would continue using the device and participants who indicated that they would not continue using the device) was carried out. Independent 2-tailed *t* tests were carried out on SUS scores for the 2 groups. In addition, correlations between question 21 and all other questions from the bespoke questionnaire were carried out using the Kendall τb rank to identify specific factors that are linked to the intention to continue using the device.

#### Predictive Modeling

A predictive model is frequently used in statistics and machine learning techniques to model the current data and predict future outcomes. For this part of the analysis, we evaluated models that may predict the intention to continue using a device after the monitoring period. These predictions were based on the usability questionnaire, where the answer to question 21 was predicted based on the answers to the other questions.

An important criterion for wearable technologies is user acceptance. This increases the likelihood that individuals will continue to use the device long-term and beyond periods when they are being actively monitored. Factors potentially influencing a user’s acceptance of a wearable device include comfort, simplicity, and device intrusiveness. For example, if a device requires frequent interaction, then it could become too much of a burden.

### Ethics Approval

Approval for the research study was obtained from each of the participating institutions where required. The Ulster University Research Governance Ethics Committee granted approval under reference REC/19/0026; the University College Cork Clinical Research Ethics Committee of the Cork Teaching Hospitals granted approval under reference ECM 4(a) 16/10/19; and the Regional Research Ethical Review Board of Umeå University, Sweden, granted approval under reference 07-031M with extensions. At the Karelia University of Applied Sciences, Finland, no ethics approval from an institutional review board was required as the research adhered to the ethical principles of research with human participants as per the Finnish National Board on Research Integrity TENK guidelines [29]. The research was conducted in accordance with the principles of the Declaration of Helsinki and in accordance with local statutory requirements. All participants provided written informed consent to take part in this study. Consent was provided for publication by all participants under the condition that the data were anonymized.

## Results

### Pretrial Questionnaire Results

Summary statistics for each of the 4 health questionnaires (SF-36, MMSE, GDS, and MDPQ) are presented in Table 1, with the general health variable selected to represent the SF-36 questionnaire and the overall MDPQ variable selected to represent the MDPQ questionnaire.

**Table 1:** Pretrial questionnaire results.

	Values, mean (SD)	Variance
SF-36^a^ general health	72.54 (18.96)	359.471
MMSE^b^	28.49 (1.55)	2.410
GDS^c^	1.43 (2.11)	4.468
MDPQ^d^ overall	3.53 (1.26)	1.577

^a^SF-36: 36-item Short Form Health Survey.

^b^MMSE: Mini-Mental State Examination.

^c^GDS: Geriatric Depression Scale.

^d^MDPQ: Mobile Device Proficiency Questionnaire.

Results show that the cohort comprised participants who were, on average, in good health as defined by SF-36 results (mean 72.54 out of 100, SD 18.96). Results showed that only 9% (6/65) of the participants scored <50 on the SF-36 general health component, implying that a small number of participants in the study perceived that they were struggling with health issues. The average MMSE value among all participants was 28.49 (SD 1.55). As previously stated, a score of <25 on the MMSE indicates some degree of dementia, whereas a score of >4 in the GDS indicates some degree of depression. Only 2% (1/65) of the participants scored <25 on the MMSE, with a score of 24. The average GDS score among all participants was 1.43 (SD 2.11), with only 9% (6/65) of the participants reporting scores of >4 in the GDS. The average MDPQ value was 3.53 (SD 1.26), which is between 3 (“not very easily”) and 4 (“somewhat easily”), indicating that our participants were between states when it comes to overall mobile phone device proficiency.

Table 2 shows the relevant background characteristics of all cohorts by region. The table includes summary statistics for age, sex, height, weight, SUS score, and bespoke usability questionnaire—questions 10 (The activity tracker was comfortable to wear at night), 17 (Using the activity tracker helped me be more active), and 21 (Would you continue to use the device and app again after the trial is finished?) and the 3 functional test scores (WT10M1, WT10M2, and STS5). The 3 usability questions are presented as device comfort and becoming more active were identified as the top 2 influencing factors for continuing to use the device. In total, 3 physical function measures were chosen: the two 10-m walking tests and the STS5 as these are deemed important measures when wearing an activity tracker.

**Table 2:** Summary of background characteristics of the participants (N=65).

Background characteristic and cohort or subcategory		Participants, n (%)	Values, mean (SD)
**Age (years)**			
	Whole group	65 (100)	70.5 (5.65)
	Finland	23 (35)	71.1 (5.98)
	Northern Ireland and Ireland	22 (34)	70.4 (7.69)
	Sweden	20 (31)	70 (0)
**Sex (female)**			
	Whole group	37 (57)	N/A^a^
	Finland	13 (20)	N/A
	Northern Ireland and Ireland	14 (22)	N/A
	Sweden	10 (15)	N/A
**Sex (male)**			
	Whole group	28 (43)	N/A
	Finland	10 (15)	N/A
	Northern Ireland and Ireland	8 (12)	N/A
	Sweden	10 (15)	N/A
**Height (cm)**			
	Whole group	65 (100)	166.9 (22.88)
	Finland	23 (35)	168.9 (8.18)
	Northern Ireland and Ireland	22 (34)	158.7 (36.42)
	Sweden	20 (31)	173.5 (9.53)
**Weight (kg)**			
	Whole group	65 (100)	72.3 (15.95)
	Finland	23 (35)	69.7 (12.55)
	Northern Ireland and Ireland	22 (34)	72.2 (20.51)
	Sweden	20 (31)	75.5 (13.81)
**SUS^b^ score**			
	Total	65 (100)	67.2 (18.27)
	Not acceptable (0≤SUS<50)	12 (18)	40 (6.99)
	Marginal (50≤SUS<70)	20 (31)	59.5 (6.57)
	Acceptable (70≤SUS≤100)	33 (51)	81.7 (9.74)
**Question 10**			
	Whole group	65 (100)	4.1 (0.92)
	Finland	23 (35)	3.8 (0.98)
	Northern Ireland and Ireland	22 (34)	4.4 (0.73)
	Sweden	20 (31)	4.2 (0.99)
**Question 17**			
	Whole group	65 (100)	3.4 (1.17)
	Finland	23 (35)	3.4 (1.08)
	Northern Ireland and Ireland	22 (34)	3.8 (1.01)
	Sweden	20 (31)	3 (1.34)
**Question 21 (no)**			
	Whole group	23 (35)	N/A
	Finland	11 (17)	N/A
	Northern Ireland and Ireland	4 (6)	N/A
	Sweden	8 (12)	N/A
**Question 21 (yes)**			
	Whole group	42 (65)	N/A
	Finland	12 (18)	N/A
	Northern Ireland and Ireland	18 (28)	N/A
	Sweden	12 (18)	N/A
**WT10M1^c^ (seconds)**			
	Whole group	65 (100)	8.0 (1.70)
	Finland	23 (35)	8.3 (1.07)
	Northern Ireland and Ireland	22 (34)	8.3 (2.52)
	Sweden	20 (31)	7.3 (0.81)
**WT10M2^d^ (seconds)**			
	Whole group	65 (100)	7.7 (1.40)
	Finland	23 (35)	7.8 (1.01)
	Northern Ireland and Ireland	22 (34)	8.3 (1.94)
	Sweden	20 (31)	7.1 (0.73)
**STS5^e^ (seconds)**			
	Whole group	65 (100)	11.6 (6.55)
	Finland	23 (35)	11.0 (1.92)
	Northern Ireland and Ireland	22 (34)	12.8 (10.90)
	Sweden	20 (31)	10.8 (2.41)

^a^N/A: not applicable.

^b^SUS: System Usability Scale.

^c^WT10M1: 10-m walk test time 1.

^d^WT10M2: 10-m walk test time 2.

^e^STS5: Five-Time Sit-to-Stand test.

### SUS Results

#### Overview

The results of the SUS questionnaire showed that the average SUS score (N=65) was 67.15 (SD 18.27). Table 3 shows the mean SUS scores for the region, sex, age, and number of wearables used.

**Table 3:** Summary of statistics of the participants (N=65).

Statistic description			Participants, n (%)		SUS^a^ score, mean (SD)		*t* test (*df*)			*P* value	
**Region**								0.091 (2)			.91
	Finland	23 (35)		68.3 (11.95)							
	Northern Ireland and Ireland	22 (34)		65.9 (19.34)							
	Sweden	20 (31)		67.3 (23.28)							
**Sex**								0.447 (63)			.66
	Male	28 (43)		65.98 (18.25)							
	Female	37 (57)		68.04 (18.50)							
**Wearables used**								0.851 (63)			.40
	Xiaomi Mi Band	28 (43)		69.4 (19.30)							
	Xiaomi Mi Band+Axivity AX3	37 (57)		65.5 (17.50)							
**Age (years)**								0.411 (2)			.81
	<70	23 (35)		71.3 (14.60)							
	70-74	32 (49)		67.6 (19.30)							
	>74	20 (31)		67.3 (23.30)							

^a^SUS: System Usability Scale.

#### Analyzing the SUS Score by Cohort Region

SUS scores can be represented using either grades or acceptability ranges. Acceptability ranges use 3 categories: not acceptable (0*≤*SUS<50), marginal (50*≤*SUS*<*70), and acceptable (70*≤*SUS*≤*100) [30]. Analysis of SUS scores by region showed that Finland had both the largest (11/23, 48%) percentage in the marginal category and the lowest (2/23, 9%) percentage in the unacceptable category, making it the best-performing region given that scores in the acceptable category were similar across regions. Northern Ireland and Ireland performed equally well in both the marginal and acceptable categories, with 41% (9/22) of people. Sweden had the largest (9/20, 45%) percentage in the acceptable category, but conversely, scored the worst in the unacceptable category (6/20, 30%). Across all regions, a total of 43% (28/65) of people thought that the device had an acceptable SUS score of >70.

**Figure 2:**
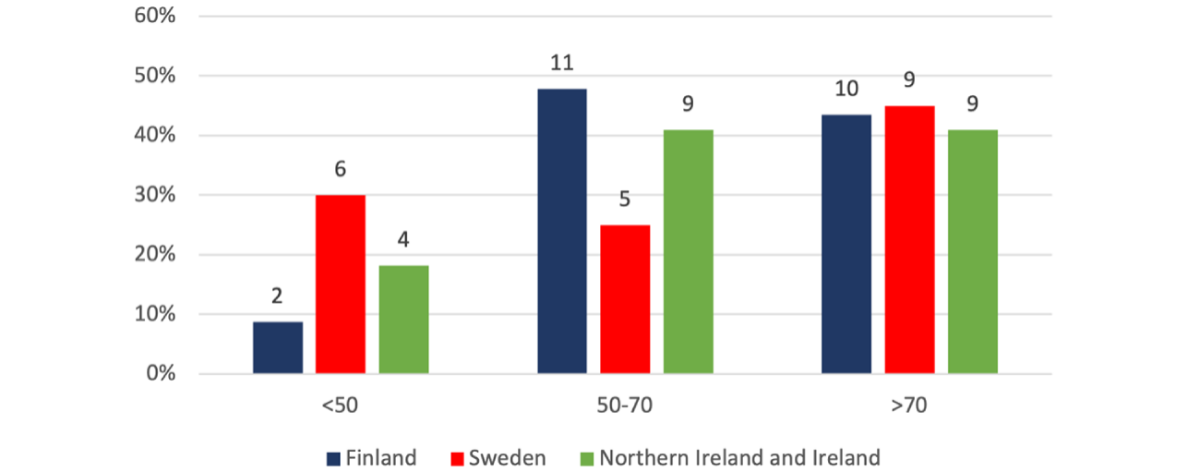
Histogram of System Usability Scale (SUS) categories from each region.

As shown in Figure 2, the distributions were not identical; therefore, a 1-way ANOVA was applied to compare the mean ranks. The results presented in Table 3 show that the differences between the mean ranks of the SUS scores for each region were not statistically significant.

**Figure 3:**
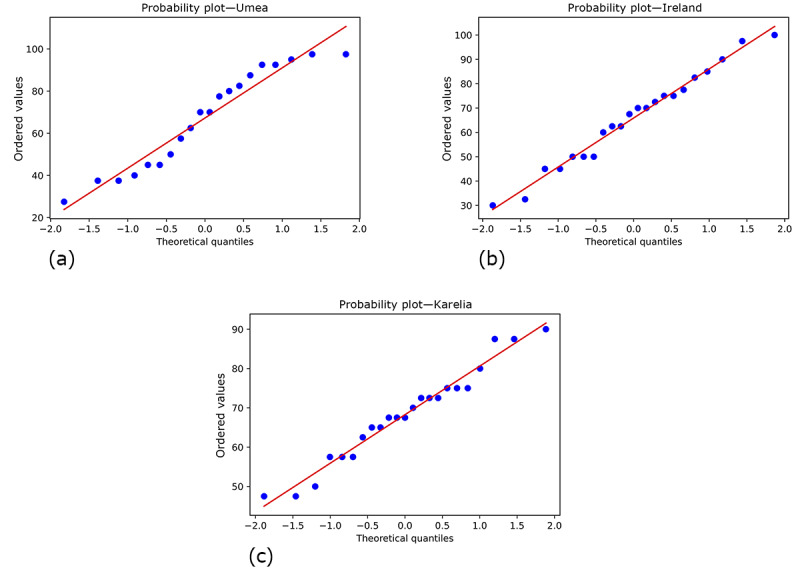
Quartile-quartile plots of the System Usability Scale (SUS) scores from each region. Plot (a) shows the scores from Umea, (b) the scores from Ireland, and (c) the scores from Karelia.

The group means were compared using a 1-way ANOVA. A Levene test was performed, resulting in a *P* value of .85; therefore, the variances can be assumed to be homogeneous, and equal variances are assumed. Observing the normal quantile-quantile plots for each region in Figure 3, the quantiles mainly lie on or close to the red line, suggesting a normal distribution.

On the basis of the results in Table 3, the means of the SUS scores for each region were not statistically significant.

#### Analyzing the SUS Score by Sex, Number of Wearables Used, and Age

An independent-sample *t* test was conducted to compare the SUS score between (1) the sexes and (2) the number of wearables used. The results suggest that there was no statistically significant difference in perceived system usability either given the participants’ sex (*P*=.66) or whether the participants wore 1 or both activity trackers (*P*=.40).

To analyze the SUS score by age, a Kruskal-Wallis *H* test was conducted, allowing for a comparison between the 3 age categories. The Levene test *P* value was <.001; therefore, the variances can be assumed to be not homogeneous. The results from the statistical test suggest that the means of the SUS scores for each age category were not statistically significant (*P*=.81).

Each of the SUS analyses showed that, regardless of comparing region, sex, wearables used, or age, there was no difference in perceived system usability.

### Bespoke Usability Questionnaire

#### Overview

**Figure 4:**
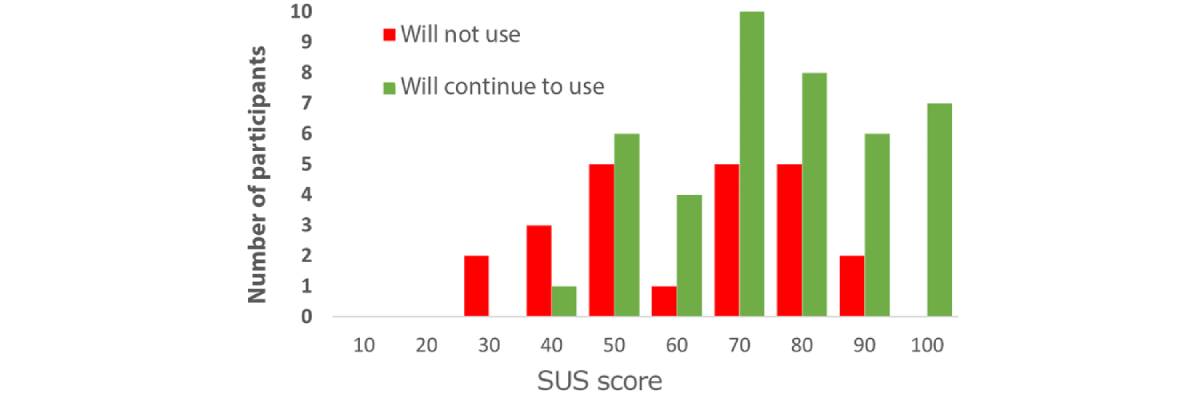
Histogram of the System Usability Scale (SUS) scores of the 2 different continued use groups.

The final question in the bespoke usability questionnaire asked the participants if they intended to continue using the device after the trial had finished. In total, 65% (42/65) of the participants said that they would like to continue using the wearable device and phone app, whereas 35% (23/65) of the participants said that they would not like to continue using the wearable device and phone app. Figure 4 shows the distribution of SUS scores for participants who intended to continue using the device compared with participants who indicated that they would not continue using the device.

To evaluate whether there was a significant difference in SUS scores between participants who indicated that they would continue using the device and participants who indicated that they would not, an independent *t* test was performed on the SUS questionnaire scores. The results are presented in Table 4. The participants who indicated that they would continue using the device averaged an SUS score of 71.8; thus, the “continue using” group on average considered the usability of the device to be within the “acceptable” category (range >70). In comparison, those who indicated that they would not be interested in continuing to use the device averaged an SUS score of 51.7 and, thus, the “not continue using” group on average ranked the usability of the device within the “marginally low acceptability” category (range >50 and <65). Results from an independent *t* test showed that the SUS scores of the 2 “continued use” groups were statistically significant.

In addition to comparing usability with the intention to continue using the device, we evaluated what effect previous activity tracker experience (usability question 2) had on usability. In total, 20% (13/65) of the participants said that they had previously used a wrist-worn activity tracker, whereas 80% (52/65) of the participants said that they had never used a wrist-worn activity tracker before the trial. Statistical significance was evaluated for the SUS score of participants who had previous experience versus those who did not have previous experience. The results from an independent *t* test (Table 5) showed that there was no significant difference between a user’s SUS score and whether they had previous experience with a wrist-worn activity tracker (*P*=.28).

Further analysis was performed on the intention to continue using the device (question 21) to evaluate how continued use was linked to other human factor and usability elements. Therefore, correlations between question 21 and the other bespoke questions were analyzed. The description of the correlation values and associated rank are presented in Table 6, where the direction of the relationship is indicated by the sign of the coefficient. The results of the Kendall τb rank correlations are presented in Table 7. The results revealed 5 usability questions that had a strong correlation with the continued device use question. Questions 10 and 17 were the top-ranking features, each with a *P* value of .003, highlighting that both comfort at night and becoming more active are key early indicators of whether a user will continue using and wearing a device.

Further analysis was performed to evaluate the possible links between participants’ physical function and continued use in the future. Two 10-m walk test measurements and a sit-to-stand test (WT10M1, WT10M2, and STS5) were compared with continued device use using the Kendall rank correlation coefficient. On the basis of question 17 (*Using the activity tracker helped me be more active*) being highly correlated with continued device use, the aim was to assess whether physical function, measured before the study period, influenced continued device use. The results of this analysis are presented in Table 8. The results showed that none of the 3 physical function measures—WT10M1, WT10M2, and STS5—correlated with continued device use. This indicates that physical function before using the device is not likely to influence whether the participant will continue using the device in the future.

**Table 4:** Summary statistics of the System Usability Scale scores for question 21 (N=65).

Continue to use the wearable device?	Participants, n (%)	Values, mean (SD)	*t* test (*df*)	*P* value
Yes, I would like to	42 (65)	71.8 (17.08)	−2.92 (63)	.005
No, I am not interested	23 (35)	58.7 (17.64)	−2.92 (63)	.005

**Table 5:** Summary statistics of the System Usability Scale scores for question 2 (N=65).

Previously worn an activity tracker?	Participants, n (%)	Mean (SD)	*t* test (*df*)	*P* value
Yes	13 (20)	72.12 (15.61)	−1.10 (63)	.28
No	52 (80)	65.91 (18.81)	−1.10 (63)	.28

**Table 6:** Kendall τb correlation ranks.

Correlation	Rank
±0.10^a^	Very weak
±0.10 to 0.19	Weak
±0.20 to 0.29	Moderate
±0.30	Strong

^a^A positive sign indicates a positive relationship, and a negative sign indicates a negative relationship. A ± value means that the correlation value can either be positive or negative for each rank (eg, a 0.15 correlation would be weak, as would a –0.15 correlation).

**Table 7:** Continued device use Kendall τb correlation for each usability question.

Question number	Question	Rank	*P* value
10	The activity tracker was comfortable to wear at night.	0.348	.003
17	Using the activity tracker helped me be more active.	0.340	.003
15	The activity tracker accurately tracked my physical activity.	0.317	.005
6	I was able to wear the device easily without help from another person.	0.308	.009
9	The activity tracker was comfortable to wear during the day.	0.306	.01
4	I think that monitoring my health 24 hours a day, 7 days a week is a good thing.	0.264	.02
5	I am comfortable with my health data being stored on the internet.	0.264	.02
13	I had no concerns about my privacy while wearing the device.	0.253	.04
2	Have you previously used a wrist-worn activity tracker before the project?	0.209	.09
14	I was happy to wear the sensor in public.	0.206	.08
8	I was able to perform my daily tasks as usual while wearing the device.	0.202	.09
18	Over the last week, how many days did you wear the device?	0.187	.12
19	Did you wear it at nighttime?	0.169	.18
16	I was happy to wear the sensor around the house.	0.164	.18
12	I was able to put on the device in a reasonable amount of time.	0.119	.31
1	Have you heard of wearable smart devices before the project?	0.115	.36
7	I was able to remove the device easily without help from another person.	0.083	.50
20	Did you remove the device during the day for reasons other than getting the device wet?	−0.078	.53
3	Did you like the appearance of the wrist-worn activity tracker?	0.054	.66
11	I was concerned that the device was not securely attached to me.	−0.019	.87

**Table 8:** Continued device use (question 21) Kendall τb correlation for each walking activity feature.

Question	Rank	*P* value
WT10M1^a^	−0.194	.06
WT10M2^b^	−0.083	.42
STS5^c^	0.057	.58

^a^WT10M1: 10-m walk test time 1.

^b^WT10M2: 10-m walk test time 2.

^c^STS5: Five-Time Sit-to-Stand test.

Some qualitative data were also recorded using the bespoke usability questionnaire. Namely, participants were asked to provide any comments on the activity tracker that were not covered by the previous 21 questions. Some participants commented that the fastening buckle used by the Xiaomi Mi Band 3 was difficult to secure at times, which is likely to have affected scoring on questions related to comfort and donning and doffing (questions 6, 7, 9, 10, 11, and 12). In addition, some participants reported that they believed that the wearable sensor had to be fastened extremely tightly to obtain an accurate reading. This factor may have also influenced their comfort perception.

#### A Predictive Model for Continued Device Use

To train the predictive model, features were chosen from the results of the Kendall τb correlation from Table 7. A total of 3 feature subsets were chosen. The first subset was based on the 2 highest-correlated features (questions 10 and 17) such that the selected features had a *P* value of ≤.005. A second subset was selected to include features with a *P* value of ≤.01 (questions 6, 9, 10, 15, and 17). Finally, a third subset was selected to include features with a *P* value of ≤.10 (questions 4, 5, 6, 9, 10, 13, 15, and 17). For the remainder of the paper, the models developed using each of the 3 feature subsets will be known as the 2-feature model, 5-feature model, and 8-feature model.

Initial experimentation was performed using multiple classifiers to obtain a performance baseline. This preliminary experimentation tested the following classifiers: decision tree, support vector machine, random forest, and k-nearest neighbor. From this experimentation, we found that random forest provided the highest predictive performance in classifying whether users would have an intention to continue using the device after the trial ended. For comparison with the random forest models, regression multinomial models were also performed. These multinomial models are helpful for the simplicity and interpretability of the predictive model.

For the multinomial models, all data were included to observe and assess the statistical or discrimination power of the model at once. For the random forest models, validation of the final classification was achieved using a train-test split validation of 70 to 30 to check model accuracy.

The results from each of the 2-, 5-, and 8-feature models for both the multinomial and random forest models are shown in Table 9.

**Table 9:** Classification confusion matrix for the 2-, 5-, and 8-feature models.

Number of features and class type			Multinomial model—predicted class			Random forest model—predicted class	
			No	Yes	No		Yes
**2 features**							
	**Actual class**						
		No	15	8	5		1
		Yes	5	37	3		11
**5 features**							
	**Actual class**						
		No	16	7	5		1
		Yes	4	38	3		11
**8 features**							
	**Actual class**						
		No	17	6	5		0
		Yes	4	38	3		12

The findings from the multinomial 2-feature model display an overall accuracy of 80%, and the findings from the random forest 2-feature model correlate with an average accuracy of 80%, an average precision of 0.80, and an average recall value of 0.80. Both sets of results show that a reasonably accurate prediction can be made for usability question 21.

After increasing the model’s features to 5, the multinomial model displays an overall percentage of 83.1%, and the random forest model correlates with an average accuracy of 80%, an average precision of 0.80, and an average recall value of 0.80. The multinomial results improved slightly compared with the findings from the 2-feature model, whereas the random forest results remained the same.

The final model used 8 parameters. The findings from this multinomial model displayed an overall percentage of 84.6%, whereas the random forest model’s average accuracy increased to 85%, displaying an average precision of 0.88 and an average recall value of 0.85. Both sets of results improved from the 2-feature and 5-feature model findings. Nonetheless, the improvement from the 2-feature model to the 8-feature model was 5%.

## Discussion

### Principal Findings

Analysis of the usability of the wearable system by older adults indicated a significant correlation between usability and intention to continue using the system. Comparing SUS scores of participants who intended to continue using the device with SUS scores of those who did not resulted in a statistically significant difference (*P*=.005). On average, users who indicated that they would continue using the wearable device also indicated that the device had good usability, whereas users who indicated that they would not continue using the wearable device indicated that the device had poor usability. Therefore, participants who found the system easier to use were also more likely to want to continue using it. These results are in line with previous research findings that suggest that ease of use and device usability are important measures for technology acceptance yet are often overlooked in favor of device accuracy [7].

Additional evaluations conducted using the standardized SUS scores showed that neither sex, age, geographical location, previous experience, nor the number of wearable devices used influenced the results of system usability. Although a subset of participants wore 2 activity trackers, the results showed no statistical difference in SUS score depending on whether the participant was asked to use 1 or 2 wearables. This is likely because 2 wrist-worn sensors are still deemed unobtrusive in everyday life. Further research is required to observe whether these results can be scaled based on anatomical location or additional wearable sensors.

Furthermore, there was no statistically significant difference in the usability scores for a wearable sensor system regardless of whether the participant had previous experience using a wearable device. This finding implies that technology literacy is not necessarily an influencing factor when it comes to the perceived usability of a wearable device. A possible explanation for this finding is that each participant in the study received a standardized training session lasting 10 to 15 minutes at the beginning of the trial and a user manual for reference. These results indicate that a lack of experience using wearable devices does not need to be a barrier to adoption if appropriate training can be provided.

Usability is clearly an influencing factor for continued device use. However, there are other human factors that could influence continued use. A 21-question bespoke questionnaire was used to further evaluate these human factors. The results showed that the human factors that had the strongest correlations with continued device use were (1) device comfort at night and (2) perception that the device helped increase activity. Inspecting the 5 questions that correlated the most with continued device use, 3 of the questions related to human factors, whereas 2 related to perceived accuracy.

On the basis of feature subsets from 8 questions that correlated the most with continued device use, machine learning models were implemented to predict whether a participant would indicate that they would continue using a wearable device. These 8 questions related to opinions on comfort, data privacy concerns, and the participants’ perception (beliefs or attitudes) of device accuracy or monitoring their health. These models have the potential to act as an early indicator of participants not continuing to use the device. Factors such as discomfort at night can be identified early before users decide to stop using it. This may allow for interventions to be made to address user concerns early in research studies, for example. As an additional benefit, the accuracy of these models provides insights into what design features are important to encourage wearable technology uptake in older adult populations.

The results indicated that the models could predict the likelihood of a participant having an intention to continue using the device with 80%-85% accuracy. Interestingly, the accuracy of the model only dropped by 5%-80% when we greatly simplified the questionnaire and only selected the top 2 correlated questions for prediction. As mentioned, these questions were related to evening comfort and whether the device helped increase activity levels. These results indicate that, by using a simple 2-question survey approach, it is possible to make accurate predictions about the likelihood of an older adult wanting to continue using a wearable device. This is useful as focus groups could leverage these questions to gain meaningful insights into their product development, or these questions could be included in a mobile app or web-based application to frequently report the usability of wearable products to ensure client satisfaction and better standards of quality assurance.

Future design of devices should keep in mind that wearable sensors are likely to be used by older adult patients with health complaints who often have reduced fine motor skills [31]. Therefore, to ensure maximum customer buy-in, manufacturers need to ensure that such devices are easy to don and doff.

### Comparison With Previous Work

Most research on wearable sensor technologies currently places accuracy at the center of the design. This often comes at the expense of usability, which can ultimately have negative effects on continued device use [6]. Previous research suggests that, to achieve successful adoption of remote rehabilitation technologies, the solution must be both practical and usable [8]. This is particularly relevant when considering wearable sensor systems.

Previous research has been conducted on usability evaluations by older adults using activity trackers [32]. This study asked 20 older adults to evaluate 5 different activity trackers over a 2-hour period. On average, the trackers tested in that study had an SUS of 56.38 (SD 11.86). Although a different cohort, review time, and number of devices were used, it is interesting to note that the Xiaomi Mi Band 3 used in our study, evaluated across 4 independent locations, obtained a similar average SUS score of 67.15 (SD 18.27) compared with the top-performing trackers in the previous study: the Fitbit Flex (SUS=66.25) and Nike FuelBand (SUS=65). SUS scores for the Xiaomi Mi Band 3 in this study were significantly higher than those of the other 3 sensors assessed by Steinert et al [30]. There is some evidence collected using posttrial interviews with some participants suggesting that the high score obtained by the Xiaomi Mi Band 3 may be related to a specific element of comfort. Participants indicated that the rubber material of the activity tracker made the device very comfortable to wear.

To achieve the potential health benefits presented by wearable sensors and remote digital health technologies for older adult populations, it is vital that users continue to use the wearable sensors over long periods. However, there is limited work in the literature exploring the factors that influence long-term wearable device use among older adults.

### Strengths and Limitations

One of the key strengths of this study is that it assessed a broad spectrum of factors that could potentially influence continued device users among a diverse set of participants. This study provides clear evidence that usability, comfort, and motivation are key elements that must be considered for any wearable sensor-based application requiring long-term use.

There are some limitations to this study that could be addressed in future research. First, the sample size was limited to 65 participants, and as such, may not be large enough to provide accurate insights into the behaviors of older adults. This limitation was imposed as data collection had to cease at the onset of the COVID-19 pandemic. Second, there may be bias in the results owing to the ethics approval granted for the study. Given that participants were to have no underlying health conditions other than frailty, the vast majority of the volunteers were considered healthy, reflected by the mean score of 72.54 achieved for general health in our cohort, calculated using the SF-36 questionnaire. To contextualize this, in a normative study on older adults (N=8117; aged ≥65 years) where no screening was imposed, the mean general health score was 53.06 [33]. The factors that influence continued device use may be different for older adults considered unhealthy. To verify this, further research with a larger cohort of both healthy and unhealthy participants would be required. Third, although the findings of this study are based on the Xiaomi Mi Band 3, it is unknown whether they would be transferable to other devices. Therefore, further research would be required administering the same bespoke usability questionnaire to older adults testing a range of wearable sensor devices.

### Conclusions

This study used a combination of validated questionnaires to gather 65 participants’ opinions on the usability of an off-the-shelf wearable sensor system, the Xiaomi Mi Band 3. To gain further insights into the factors that may influence an older adult intending to continue using a wearable device, we also designed a bespoke usability questionnaire for this study. Various analyses were performed examining the statistics from the pretrial questionnaires; summary statistics of the SUS score with respect to region, sex, wearables used, and age; and findings that focused specifically on the final question from the bespoke usability questionnaire to determine what factors influence continued device use.

The results from the SUS show that there was no notable difference in perceived system usability depending on region, sex, age, or previous experience, eliminating the notion that usability perception differs based on geographical location, sex, or deviation in participant age. Previous studies have suggested that usability and ease of use are as important as device accuracy when it comes to technology acceptance and device uptake. One of the main lessons learned from the results of this study was that the most important factor that influenced continued device use in an older adult cohort was device comfort. Feeling that the device was fit for purpose (ie, it helped them achieve the task it claimed it would) was the second most important factor. In addition, it was observed that comfort matters the most when a wearable device is used while sleeping. These lessons could better inform the design of future wearable sensor systems for applications specifically targeting older adults.

We presented a random forest model with 80% accuracy using these 2 features, which could be used as an early identifier of continued device use—for example, if the user is asked these 2 questions after the first day of the study, their response would be a clear sign of whether they are interested in using a wearable sensor system long-term. After including the top 8 ranked questions from the bespoke questionnaire as features of our model, the accuracy increased to 88%.

## Appendix

Multimedia Appendix 1 Accuracy, feasibility and acceptability of wireless monitoring in older people: posttrial usability questionnaire.

## Abbreviations

GDS: Geriatric Depression Scale
MDPQ: Mobile Device Proficiency Questionnaire
MMSE: Mini-Mental State Examination
SF-36: 36-item Short Form Health Survey
STS5: Five-Time Sit-to-Stand test
SUS: System Usability Scale

## Data Availability

The data sets generated and analyzed in this study are not publicly available as the data contain information that could compromise the privacy of the research participants. The data are available from the corresponding author upon reasonable request.
