# Supplementary material for: Factors Influencing Continued Wearable Device Use in Older Adult Populations: Quantitative Study
Source: JMIR Aging. 2023 Jan 19;6:e36807. doi: 10.2196/36807 (PMC9947821; doi:10.2196/36807)
Supplement: Multimedia Appendix 1 [file aging_v6i1e36807_app1.docx]

**Accuracy, feasibility and acceptability of wireless monitoring in older people**

Wearable devices

1. Had you heard of wearable smart devices before the project? Yes  No 
2. Have you previously used a wrist worn activity tracker before the project? Yes  No 
3. Did you like how the appearance of the wrist worn activity tracker?

Yes  No 

| **Statement** | **Strongly Disagree (a)** | **Disagree**  **(b)** | **Neutral**  **(c)** | **Agree**  **(d)** | **Strongly Agree**  **(e)** |
| --- | --- | --- | --- | --- | --- |
| 1. I think that monitoring my health 24 hours a day, 7 days a week is a good thing |  |  |  |  |  |
| 1. I am comfortable with my health data being stored on the internet |  |  |  |  |  |
| 1. I was able to wear the device easily without help from another person |  |  |  |  |  |
| 1. I was able to remove the device easily without help from another person |  |  |  |  |  |
| 1. I was able to perform my daily tasks as usual while wearing the device |  |  |  |  |  |
| 1. The activity tracker was comfortable to wear during the day |  |  |  |  |  |
| 1. The activity tracker was comfortable to wear at night |  |  |  |  |  |
| 1. I was concerned that the device was not securely attached to me |  |  |  |  |  |
| 1. I was able to put on the device in a reasonable amount of time |  |  |  |  |  |
| 1. I had no concerns about my privacy while wearing the device |  |  |  |  |  |
| 1. I was happy to wear the sensor in public |  |  |  |  |  |
| 1. The activity tracker accurately tracked my physical activity |  |  |  |  |  |
| 1. I was happy to wear the sensor around the house |  |  |  |  |  |
| 1. Using the activity tracker helped me be more active |  |  |  |  |  |

**General Questions**

1. Over the last week, how many days did you wear the device? ______
2. Did you wear it at night-time? Yes  No 
3. Did you remove the device during the day for reasons other than getting the device wet? Yes  No 
4. Would you continue to use the device and app again after the trial is finished? Yes  No 

Do you have any comments about the tracker device?

**Thank you for taking part in this study and for your time in completing this questionnaire**
